# Internationalization of traditional Chinese medicine: current international market, internationalization challenges and prospective suggestions

**DOI:** 10.1186/s13020-018-0167-z

**Published:** 2018-02-09

**Authors:** Annie Xianghong Lin, Ging Chan, Yuanjia Hu, Defang Ouyang, Carolina Oi Lam Ung, Luwen Shi, Hao Hu

**Affiliations:** 1 International Research Center for Medicinal Administration, Peking University, Beijing, China; 2 State Key Laboratory of Quality Research in TCM, Institute of Chinese Medical Sciences, University of Macau, Room 2057, Building N22, Avenida da Universidade, Taipa, Macao

**Keywords:** TCM, Internationalization, International market, Quality control, Standardization

## Abstract

Through reviewing the current international market for traditional Chinese medicine (TCM), this paper identified the internationalization challenges for TCM, including unclear therapeutic material basis and mechanism, difficulty of quality control, low preparation level, registration/policy barriers, and shortage of intellectual property. To deal with these challenges, suggestions were given including: (1) product innovation of TCM (study the TCM by using the methods and means of western medicine; innovate the basic theory of TCM; develop TCM health product); (2) standard innovation of TCM; (3) building big data platform of Chinese medicine (big data platform of TCM preparation; big data platform on the quality of TCM).

## Background

Traditional Chinese medicine has an extensive history and, for 5000 years, has been the fruit of the Chinese people’s intelligence in the struggles against diseases. With the advancement in medicine and technology, and the quest to better understand the science of natural medicine, the interests in and acceptance of traditional Chinese medicines (TCM) continues to grow. Featured with the long history of use in treating certain challenging diseases resulting in curative effects, comparatively lower costs and reportedly less side effects, the role of TCM in health is widely recognized around the globe [1].

In 1996, China newly introduced the concept of “internationalization of TCM” which comprised of two major aspects: (1) it is important to expand the volume of import and export of TCM in order to push forward the “going abroad” of TCM, to promote the sustainable development of TCM international trade and to foster the TCM market share across the countries; (2) the legal status of TCM in overseas countries has to be appropriately established to ensure reasonable market entry and to enable sustainable development of TCM under the protection of the local laws and regulations [2].

After more than 2 decades of commitment to the internationalization of TCM nationwide, the progress appears to be stagnated. In order to formulate strategies to achieve a break through, the obstacles inhibiting the internationalization of TCM need to be reviewed systematically. However, limited studies on the challenges facing the internationalization of TCM, this paper aims to study the current international market for TCM, to analyze internationalization challenges, and to propose solutions that can help facilitate the internationalization of TCM in the future.

## Analysis of the current international market for TCM

Internationalization of TCM has seen a decline in the volume and value of export. According to the China Customs statistics, the import and export value of TCM in 2016 experienced a negative growth for the first time in nearly 10 years despite a slight increment in the import and export value of Chinese pharmaceutical products compared to 2015. The total value of TCM import and export in 2016 was 4.6 billion USD, accounting for 4.45% of the total value of the import and export of Chinese pharmaceutical products. While the export value of TCM in 2016 dropped by 9.13% compared to 2015 from 3.77 to 3.426 billion USD, the import value achieved a big reversal, increasing 14.50% from $1.025 billion in 2015 to 1.174 billion USD in 2016.

TCM encompasses a wide range of items: traditional Chinese medicinal materials, decoction pieces, Chinese patented medicines, herbal extracts, and health care products. The export volume and values of most of these items have declined over the years. For instance, the export value of Chinese medicinal materials and decoction pieces in 2016 was 1.025 billion USD, a 3.13% decrease in growth rate year-on-year. For the health care products, the export value in 2016 was 249 million USD which was a 13.22% reduction from the same period of the previous year. The export value of the Chinese patent medicines was 225 million USD in 2016 which is equivalent to a decrease by 13.94% as compared with the previous year with the export volume declined greatly. The most significant impact on the declined volume and value of export came from the Chinese herbal extracts. In the past, these extracts were on high demand as the market for new products of essential oil bloomed both nationally and overseas. The Chinese herbal extracts were the largest and fasting growing item among all Chinese pharmaceutical products in 2015, accounted for the largest share of the total export of CM. While the export value of Chinese herbal extracts accounted for 56.2% in 2016, it failed to maintain a growing trend. The export value was only 1.972 billion USD in 2016 which was a decrease of 10.93% compared to that of last year, and the export volume also decreased by 14.3% [3, 4]. The significant downfall in the export value of herbal extracts greatly contributed to the overall negative growth of TCM export.

TCM were exported to 185 countries and regions and its main markets still remained in Asia with the dominant target markets located in Hong Kong, Japan, Malaysia, South Korea and Indonesia. Next to Asia was the United States. In 2016, China exported 526 million USD worth of TCM to the United States, accounting for 15.34% of TCM exports for China. It is anticipated that the United States would soon overtake Hong Kong and Japan and became the largest market of TCM exported from China [5]. Although TCM has a large, promising world market, it also has many obstacles. TCM’s entry into the American market requires the approval of the Food and Drug Administration (FDA), which implies large capital (over 100 million USD) and lengthy process (lasting at least 8 years). However, the more stinging problem in the entry process is that the risks of American financing support for TCM enterprises implementing internationalization strategy are still neither clear nor strong enough [6]. With the implementation of “Regulations for Botanical Drug Approval” and “Guidance for Industry: Botanical Drug Development”, the United States began to consider botanical drug compounds as a therapeutic drugs. Take “Xuezhikang (XZK)” for an example, with the special fund support for innovation and preparation of major new drugs designated in the 12th 5-year of China, it entered the FDA clinical trial. This is the first time for domestic clinical organizations to participate in registration and clinical study of lipid regulating TCM in FDA. For “Xuezhikang (XZK)” to enter clinical phase successfully, a good clinical trial protocol should be well prepared and the proper indications with supplementary functions to be tested in the clinical trials should be well selected [7].

The drop of the two major markets in Hong Kong and Japan is another major reason for the decline in the overall TCM export value and export volume. Japan was once the largest importer of Chinese medicinal materials and mainly involved Chinese medicinal materials and decoction pieces [8]. But after 1990, Japan began to vigorously develop the herbal medicine base and establish the herbal plantations in order to become self-sufficient of medicinal herbs and to reduce import from China, which led to Japan’s falling import of volume and value of Chinese medicinal materials. In 2016, the market began to pick up, embodied in the growth of export volume and value. Today, Japan has become the second largest market for TCM exports next to the United States, with an export value of 505 million USD in 2016, a 6.62% increase over the past year. There are more than 200 Japanese Kampo medicine manufacturers in Japan. These domestic enterprises possess advanced equipment and technology to process Chinese medicinal materials and to manufacture quality-stable TCM under strict standards. The locally manufactured TCM has occupied a considerable share of the markets of Chinese patent medicines and health care products. In South Korea, the drop in the sales of TCM manufactured in China was multifactorial: the issues with the packaging design and promotion strategy of TCM discouraging the use and masking the true value of TCM in health; and concerns over the content of heavy metals and pesticide residues exceeding the national standards, which are a lot more stringent compared to standards alike in many developed countries [9]. In addition, many developed countries have set up high green trade barriers and technical trade barriers for TCM entering their countries, hindering the process of internationalization of TCM.

Another factor discouraging the internationalization of TCM is the high policy thresholds. In certain countries such as Russia, Vietnam and Australia, TCM is sold as a pharmaceutical drug. In many other countries and regions, however, TCM are approved for sales as health care product, active pharmaceutical ingredient (API), or dietary supplement. On 1st May 2011, the European Union’s 2004/24/EC EU Directive on traditional herbal medicine (namely the EU botanical drug approval and continuity clause) came into force. This directive of the European Union provides a simplified registration procedure for traditional herbal medicines, and gives a 7-year transition period of the traditional herbal medicine sold in the EU market before 2004. However, after 7 years, all unregistered traditional herbal medicines will be removed from the EU market [10]. At present, Diaoxinxuekang capsule, which is developed and manufactured by Chengdu Diao Pharmaceutical Group Company, is the only one China-manufactured traditional herbal medicine marketed and sold in the EU market. It is approved as the secondary-types of TCM new drugs and carries Chinese independent intellectual property rights. This exceptional product helped demonstrate how a high policy threshold is translated into great obstacles to the entry of TCM into the international market, greatly reducing the export volume of TCM in the EU market especially the British market.

## Internationalization challenges for TCM

The therapeutic material basis and mechanism of TCM are key to the quality, effectiveness and safety of TCM, and are the fundamental prerequisite for the internationalization of TCM. The effective substance basis of TCM is the general name of chemical components that bring about the clinical efficacy of the medicine. Due to the diversity and complexity of compound structures, a TCM is a natural “combinatorial chemical sample library.” There are more than 200,000 known compounds in TCM, but currently only a few compounds such as artemisinin and arsenious acid have been studied thoroughly and clearly for their pharmacological action and biological mechanism. The targets and biological mechanisms of most compounds still remain unknown.

On the other hand, each TCM consisting of multiple compounds is a complex chemical system. Its characteristics of synergistic reaction of multi-components, multi-channels and multi-targets are difficult to be elucidated by the study model of “single component and single target” in modern medicine. The uncertainties in the effective substance basis and the mechanism of TCM further make it difficult to formulate a scientific and effective system for evaluating the efficacy and safety of TCM. International communities always find it hard to recognize and adopt the quality standards and the standards of production management of TCM. As a result, Chinese patent medicines lose their competitiveness the international market [11].

The quality control of TCM is directly related to the safety and effectiveness of TCM and is critical to the internationalization of TCM. China has set up a quality inspection technology system focusing on chemical marker detection. This method has gradually developed into quality analysis mode of quantitative analysis, chromatographic fingerprint, and discriminant analysis from the initial sensory experience. However, the research pattern of the quality control of TCM is still relying on the analysis method research. This requires careful determination of focusing on the quality inspection of Chinese traditional medicine and ignoring the quality control during the production process; paying attention to the chemical markers detection and ignoring the biomarkers; and emphasizing the drug quality analysis and evaluation and ignoring the study on the medicines regulation system. The scientific and reliable quality assurance system complying with the characteristics of TCM are yet to be fully constructed [12]. Meanwhile, assurance of TCM safety is also a difficult task added to the quality control of TCM. Many countries have added test items of microbes, preservatives, pesticide residues, heavy metals and aflatoxin for traditional medicines and herbal health foods, and formulated their national standards presenting significant cross-country variations. Although China has strengthened the quality control in microorganisms, pesticide residues, heavy metals and arsenic salts, there is still a large gap between Chinese and international standards.

The low level of dosage form is one of the important factors that restrict TCM internationalization. The modernization level of the TCM formulation is relatively backward. Tablets, capsules and granules accounted for more than half of current TCM dosage form. The advanced dosage form for TCM such as sustained-release, controlled-release and targeted preparations are still in the research stage. The majority of small and medium-sized TCM enterprises still use the traditional excipients, which makes it difficult for the TCM formulation to meet the international standard requirements. The basic research on TCM formulation is backward, especially the researches on pharmacokinetics, stability, dose–response relationships, etc. are relatively scarce.

The internationalization of TCM is also faced with a number of registration/policy barriers. Along with the gradual expansion of the international market of natural medicines, some developed countries have implemented trade protectionism in order to protect the interests of the domestic pharmaceutical companies and related medical services, set up a multitude of technical barriers to trade and continue to strengthen the management measures of the import Chinese patent medicines, which seriously hampered the process of the internationalization of TCM. These technical barriers to trade are various, such as technical standard barriers, technical regulation barriers, patent technology barriers, green barriers, etc., which makes Chinese products fail to enter or forced to withdraw from the target market due to shortcomings in technology, environmental protection, and other areas mandated by the policy and regulations.

The intellectual property of TCM is also the weak link of the internationalization of TCM. There are multiple issues related to the patents of TCM: low international patent application, low effective patents, numerous invalid patents, low patent quality and fast growth of application volume not transformed into a rapid growth of market share and so on [13]. At the same time, there are also many problems in the technology transfer of TCM, including lacking of original innovation, lacking of basic research, serious shortage of R&D investment, dislocation of R&D subjects, imperfect market transformation and industrialization mechanism, and weak service links.

## Suggestions for further internationalization of TCM

The cross integration of multidisciplinary and innovative drug research and development have brought new opportunities for the development of TCM [14].

### Product innovation of TCM

#### Study TCM by using the methods and means of western medicine

With the continuous development and progress of modern science and technology, the technology of quality identification, extraction and separation, analysis and detection of TCM is becoming more and more mature, and its action of mechanism and active components are becoming increasingly clear. In addition, the modern chemistry, biology, physics, information science and other disciplines have continuously permeated the field of research and development of TCM, which provides a new model for the research and development of TCM [15]. Academician Zhang once suggested that “the development of new varieties of TCM should focus on the research and development of TCM with high technology content such as component medicine and drug monomers, etc.; special attention should be paid to the research of new formulations of Chinese herbal preparation; attempts should be made to achieve breakthrough on traditional dosage forms such as bolus, powder, plaster, and pellet, and develop new formulations with high bioavailability, convenient administration and high level of quality control” [16]. In addition to the innovative research and development of monomeric drugs and dosage forms, the methods and means of western medicines can also be adopted in the research of TCM prescription as deemed appropriate. Prescription of TCM is not just a combination of TCM ingredients. There are new ingredients generated in the formation of prescriptions, which warrants thorough analysis of the active components and the extractions. This is considered conducive to the rapid development of TCM research and development [15].

#### Innovate the basic theory of TCM

TCM has its own connotations and it is not simply equivalent to botanical drug. If the TCM pursues westernization and abandons the theory of TCM, the research road of the TCM will be narrower and narrower [17]. Different from the western medicine which starts from the molecular biology, TCM is researched and developed based on systematic biology. After a long time of use, the toxicity and efficacy of TCM becomes clearer [18]. Rather than exploring from the very beginning and exploring pharmacological action from pharmacological experiments, the most important thing in TCM research and development is to explore and utilize ancient medicine literatures and thousands of years of clinical experience.

The improvement of clinical research of TCM can also drive the innovation and development of TCM. This provides a ground where the concepts and methods of evidence-based medicine, the principles of international clinical research, and the characteristics of TCM clinical research can all be combined. In recent years, sufficient experiences in the exploration of the clinical research of TCM has been accumulated for the design of measures that promote the improvement of the overall levels leading to many landmark achievements. For example, apricot Shi Gan Tang with Yinqiao powder for the treatment of H1N1, etc. [19].

In order to demonstrate the scientific, technological and economical values of the TCM industry for its sustainable development, academician Zhang introduced the concept of the secondary development of TCM. This concept refers to the re-development of the TCM products which have good clinical efficacy and significant market share in order to promote the redesign of the TCM product, accelerate the cultivation of famous Chinese medicine varieties and develop China’s own “blockbuster” drug [20]. With the help of modern science and technology, the development of a safe, effective, stable and controllable compound Chinese medicine is promising [21].

#### Development of TCM health product

Globally, one of the sunrise industries pivot towards health care products, which are referred to as “healthcare food”, “functional food” and “food supplement” in different countries. With the 1000 years of usage history, the TCM healthcare products have huge international market potential and room for expansion [22]. Healthcare products of TCM provide a great potential route for TCM to enter the international market. Chen Shilin pointed out that “TCM has the homology tradition of medicine and food”. Among them, many Chinese herbal medicine and decoction pieces can enter the market as health products. However, the TCM are facing many challenges including export volume decreasing, uneven distribution of export areas, and less proportion of developed regions such as in the US and EU as well as lacking of brands winning the trust of the customers” [23]. The industry of TCM health products should strive to establish a sound quality management system, seek protection of the processing technology, establish quality identification and intellectual property rights, and select appropriate promotion and sales strategy [24].

### Standard innovation of TCM

In order to enter the international market, the key step of promoting TCM is to optimize our own standard to make it acceptable to the international market [25]. The important method is to include the standard of TCM in the national pharmacopoeias such as the US Pharmacopoeia or the European Pharmacopoeia. More than 140 countries in the world have adopted the US pharmacopoeia, while the European Pharmacopoeia standard is widely used among all the European Union country members. Being included in these two Pharmacopoeia indicates a global acceptance of the TCM standards which will greatly benefit the internationalization of these products. In recent years, 27 standards of “9 varieties” such as salvia miltiorrhiza, pseudo-ginseng and ganoderma have been included in the US Pharmacopoeia, and a number of standards have entered the US Pharmacopoeia audit procedure [19]. More than 10 TCM standards including uncaria and platycodon entered the European pharmacopoeia or forum. These achievements have proved to the world that TCM can also be evaluated according to international standards, which brings new prospects for the entry of TCM into the international market.

### Building big data platform of TCM

#### Big data platform of TCM preparation

A systematic and transparent preparation of large data information platform of TCM preparation is key to comprehensive mining of the vast amounts of information on TCM and its preparation field, professional processing of data—drawing of scientific knowledge map, tracing patent data, digging and analyzing of clinical trial data and marketed drug data, drawing the navigational chart of TCM and its preparation in the era of big data. The establishment of the platform will effectively integrate all aspects of TCM research and development, serve the needs of multiple demand groups including enterprises, government regulators, academic research institutions, and so on, and greatly shorten the development time of new varieties of Chinese medicine and its preparation.

#### Big data platform on the quality of TCM

The quality data platform of TCM includes the quality standards of Chinese medicinal materials, decoction pieces, and Chinese patent medicine, so as to enhance the research on the technical standards and specifications of varieties, quality, planting, collecting, processing, preparing of decoction pieces and extraction of TCM. For example, the DNA barcode species identification system that was jointly established by the National Key Laboratory of TCM quality in Macao University and Beijing Union Medical College won the second place of the 2016 National Science and Technology Progress Award. This barcode species identification system has established a “genetic identity card” for TCM. The achievement also established the world’s largest TCM DNA barcode identification database, which contains more than 1.70 million DNA sequences, and may achieve rapid identification to almost all herbal species included in CP, USP, JP, EP, South Korea Pharmacopoeia and India Pharmacopoeia, etc., and promote TCM identification study to enter into the standardized gene identification era.

## Concluding remarks

To promote the internationalization of TCM, the government, academic institutions and industry must cooperate closely, and establish an international platform for the development and communication of TCM rather than just the domestic efforts limited in China. The establishment of the international cooperation platform is not only conducive to the internationalization of TCM but also provides opportunities for other countries and regions to develop traditional medicines, so as to better promote the traditional medicines to serve the global health.

## Abbreviations

TCM: traditional Chinese medicine
FDA: Food and Drug Administration
API: active pharmaceutical ingredient
